# Examining the Potential Mental Health Costs of Defending Victims of Bullying: a Longitudinal Analysis

**DOI:** 10.1007/s10802-021-00822-z

**Published:** 2021-04-14

**Authors:** Sarah T. Malamut, Jessica Trach, Claire F. Garandeau, Christina Salmivalli

**Affiliations:** 1 Department of Psychology, INVEST Research Flagship, University of Turku, Turku, Finland; 2 Behavioural Science Institute, Radboud University, Nijmegen, Netherlands

**Keywords:** Defending, Depressive symptoms, Social anxiety, Victimization, Popularity, Bullying

## Abstract

It has been speculated that defending victims of bullying is stressful for youth, and may contribute to poor mental health among those who regularly intervene to defend their victimized peers. However, the extant literature is thus far primarily limited to correlational, single-informant studies. The current study examined the concurrent and prospective mental health costs (e.g., social anxiety, depressive symptoms) of peer-reported defending among 4085 youth (43.9% boys; *M*_age_ = 14.56, *SD* = 0.75). Moreover, we examined two potential moderators (victimization and popularity) of the association between defending and internalizing problems. Analyses revealed that there was no evidence of a direct, positive relationship between defending and internalizing symptoms. However, a positive, concurrent association was found between defending and social anxiety, but only among youth who reported that they were also victims – the association was negative among non-victimized youth. In addition, both peer-reported victimization and social status were found to moderate the longitudinal relationship between defending and later symptoms of depression. Specifically, among low-status highly victimized youth, defending was associated with an increased risk of experiencing symptoms of depression, whereas high-status youth who were rarely seen as victims reported decreased symptoms of depression at T2 if they also had a reputation for defending others. The findings suggest that defending others is likely not a risk factor for youth who are not already vulnerable and/or have the protection of high status, and may actually have a protective effect for these youth.

Bullying is a serious problem that has negative implications for victims’ social and emotional health and well-being (see McDougall & Vaillancourt, 2015). Although compelling evidence suggests that encouraging youth to intervene and defend victims might be an effective way to reduce bullying (e.g., Hawkins et al., 2001; Salmivalli et al., 2011) and improve outcomes for victims (Ma & Chen, 2019; Sainio et al., 2011; Williford et al., 2012), there has been speculation that defending a victimized peer is a potentially risky behavior that may contribute to poorer mental health outcomes among youth who defend (e.g., Lambe et al., 2017). Although defending has been linked with several indicators of positive adjustment among youth, including higher self-esteem, greater peer acceptance and social support, and elevated social status (e.g., Lambe et al., 2019; Ma et al., 2019; Pöyhönen et al., 2010; van der Ploeg et al., 2017), several cross-sectional studies have identified a positive association between defending and youth reports of psychosomatic and internalizing problems (Callaghan et al., 2019; Jenkins & Fredrick, 2017; Lambe et al., 2017). Yet due to a dearth of longitudinal studies, it remains unclear whether defending actually places youth at increased risk for developing mental health difficulties. To address this notable gap in the research literature, we first sought to clarify previously observed concurrent associations between defending and internalizing symptoms (i.e., social anxiety and depressive symptoms). We then extended this work to examine whether having a reputation for defending could be linked to the development of internalizing problems. Moreover, we examined two possible moderators (victimization and popularity) that were hypothesized to influence whether defending was related to current and future internalizing symptoms.

## Defending & Internalizing Symptoms

There is increasing recognition of the power of bystanders to either support or discourage bullying among their peers (Hawkins et al., 2001; Kärnä et al., 2010; Menesini et al., 2015; Saarento et al., 2015; Salmivalli et al., 2011; Thornberg & Wänström, 2018). Nevertheless, some concerns have been raised about the possible negative ramifications of defending for those who intervene. According to the social-ecological diathesis stress model of bullying (Swearer & Hymel, 2015), involvement in bullying is a negative life event that may contribute to psychopathology (e.g., internalizing and externalizing problems), depending on an individual’s personal characteristics, life experiences, and the quality of their social environments. Indeed, there is growing evidence that simply witnessing bullying is associated with increased psychological distress among bystanders (e.g., Callaghan, et al., 2019; Janosz et al., 2008; Lambe et al., 2017; Rivers et al., 2009). However, it is not yet clear whether youth who actually stand up for victims experience poorer mental health outcomes.

At present, only a few studies have investigated the association between defending victims of bullying and the presence of internalizing problems among youth. For example, compared to passive bystanders, youth who defend are more likely to report experiencing negative emotions like guilt (Mazzone et al., 2016; Pronk et al., 2016) and anger (Lambe et al., 2017; Trach & Hymel, 2019) while witnessing bullying. Similarly, after controlling for demographic characteristics and previous involvement as a bully or victim, bystanders who reported helping a victim of bullying in the past 2–3 months also reported experiencing elevated psychological symptoms, whereas doing nothing in response to witnessed bullying was not associated with any mental health symptoms (Callaghan et al., 2019). Self-reported defending has also been positively associated with concurrently measured internalizing symptoms (Evans et al., 2019; Jenkins et al., 2017; Jenkins & Frederick, 2017; Wu et al., 2016). Still, other researchers found defending to be associated with lower school-related anxiety (Correia et al., 2009) and higher self-esteem and optimism for the future (Evans et al., 2019). Thus far, only one other study has investigated the association between defending and internalizing symptoms using a longitudinal design (Duomas et al., 2019). This study evaluated the effects of a brief bystander intervention designed to train student leaders to act as defenders, with the result that female adolescents who completed the training reported significant decreases in internalizing symptoms at follow-up, compared to those in the control group. However, this study did not consider actual rates of defending or changes in bystander behavior as a result of the intervention in relation to youth’s mental health. The consequences of being trained to act as a defender in a controlled setting (e.g., feeling empowered) may differ compared to defending spontaneously in real cases of bullying (e.g., feeling vulnerable or exposed).

Taken together, the current literature on the prospective link between defending and internalizing problems is scarce and inconsistent. Given the growing call in the field to encourage youth to defend their peers, it is essential to examine whether defending is in fact positively or negatively associated with internalizing problems, and whether youth are at risk of experiencing elevated internalizing problems as a consequence of defending. Much of past research has used self-reports for the assessment of both defending behavior and internalizing problems, which raises the issues of social desirability bias for defending as well as common method bias. Finally, previous studies failed to account for potential moderators of the relationship between defending and internalizing problems, which may help to clarify for whom defending is either positively or negatively associated with internalizing symptoms. The current study posits that the association between defending and internalizing symptoms will depend on youth’s other characteristics – namely, their victimization experiences and popularity.

## Victimization and Popularity as Potential Moderators of Defending and Internalizing Problems

According to interpersonal risk models of internalizing difficulties (e.g., Epkins & Heckler, 2011; Rudolph et al., 2008), youth’s social relationships play an important role in the development of psychopathology. Peer relationships are a crucial aspect of youth’s development (e.g., Brown & Larson, 2009), and negative experiences in this domain, such as victimization by peers, can be viewed as an indication of youth’s vulnerability and a risk factor for maladjustment (e.g., Troop-Gordon, 2017). Indeed, a meta-analysis found strong support for peer victimization as a predictor of future internalizing problems (Reijntjes et al., 2010). In addition, Epkins and Heckler’s (2011) *cumulative* interpersonal risk model suggests that the presence and interaction of multiple sources of risk contribute to negative adjustment. Thus, if defending may put youth at risk for internalizing symptoms (Callaghan et al., 2019; Lambe et al., 2017), then these mental health concerns would likely be exacerbated by other interpersonal difficulties, such as victimization. In other words, defending may only be associated with adverse consequences for youth who are already vulnerable, such as youth who are victimized *(defender vulnerability hypothesis).* This perspective is partially supported by findings that being a victim of bullying moderated the association between witnessing bullying and depressive symptoms (but not anxiety; Midgett & Doumas, 2019). A similar association may be true for defending (rather than simply witnessing bullying), yet much of the extant literature has not accounted for the potential overlap between defending and victimization. As self- and peer- reported victimization are differentially associated with adjustment (Scholte et al., 2013), the current study will consider the possible moderating effects of both self- and peer- reported victimization to examine similarities/differences across reporting methods and to account for potential shared variance biases.

In contrast, popularity may help to mitigate any potential risks of defending on youth’s mental health (*defender protection hypothesis)*. That is, a certain level of status may be required for youth to defend others without experiencing adverse consequences. Indeed, youth who are nominated as defenders also tend to be seen as popular and well-liked by their classmates (Lambe et al., 2019; Ma et al., 2019). Thus, it has become a widespread belief that their elevated social status offers defenders the social capital needed to intervene safely, or with minimal consequences. However, the effect of popularity as a moderator of the association between defending and negative adjustment has not been empirically tested. Popularity is generally viewed as an indicator of social competence that is associated with social benefits, and therefore may be protective against psychological maladjustment (e.g., Sandstrom & Cillessen, 2006, 2010). On the other hand, high levels of popularity have also been associated with an assortment of risks for adolescents (e.g., Schwartz & Gorman, 2011). Specifically, high (and low) levels of popularity are both associated with poorer psychosocial adjustment, compared to youth with moderate levels of status (Ferguson & Ryan, 2019). Nevertheless, whether or not defending is associated with internalizing problems is likely related to whether youth have the social resources to defend their peers. In other words, defending may be less stressful for youth if they are well-connected in the peer group and have peer support (i.e., high popularity). Therefore, defending was expected to be positively associated with internalizing symptoms for youth with low levels of popularity, and negatively associated with internalizing symptoms for youth with high levels of popularity.

In addition to the independent effects of victimization and popularity on the internalizing symptoms associated with defending, there may be an additive effect of these two variables. For example, a common narrative in the literature is that some youth are victimized precisely because they are unable to defend themselves (e.g., Troop-Gordon, 2017). Accordingly, it is possible that youth who are high in both defending and victimization lack the necessary social skills and/or resources to defend successfully or without consequences (e.g., receiving negative feedback from peers), and subsequently experience higher levels of internalizing problems. Thus, youth who are high in defending and high in victimization, but low in popularity, may be even more likely to develop internalizing problems. However, it is important to note that youth with high levels of popularity can also be targets of aggression from their peers (Dawes & Malamut, 2018; Malamut et al., 2020). In fact, high-status youth who were victimized were found to be more likely to increase in internalizing symptoms 6 months later compared to lower-status victims, as they have “more to lose” (Faris & Felmlee, 2014). Therefore, it is also possible that youth who are high in popularity, frequently bullied, and who often defend others may also experience elevated internalizing symptoms. In light of these considerations, the current study will also explore whether there is a three-way interaction between defending, victimization, and popularity in predicting internalizing symptoms.

## The Current Study

Despite growing speculation that defending victimized peers may be a risky endeavor for youth (e.g., Lambe et al., 2017; Meter & Card, 2015), the extant literature is inconclusive due to mixed findings from cross-sectional research and a dearth of longitudinal research. Given the possible benefits of defending on victims’ adjustment (Ma & Chen, 2019; Sainio et al., 2011) and bullying prevalence in the peer group (Salmivalli et al., 2011), it is essential to examine whether defending does in fact put youth at risk for adverse outcomes. To this end, the current study will build on past research in several key ways. First, the current study will replicate past studies using a cross-sectional design, while controlling for other variables (i.e., victimization, popularity) that may influence the association between defending and two forms of internalizing problems (social anxiety and depressive symptoms, analyzed separately). Second, we will examine the effect of defending on the development of internalizing symptoms 5 months later. Lastly, we will test whether victimization and popularity moderate the concurrent and prospective association between defending and internalizing symptoms. Specifically, defending was hypothesized to be associated with higher internalizing symptoms at high levels of victimization and with lower internalizing symptoms at high levels of popularity. Furthermore, the current study explored whether there was a three-way interaction between defending, victimization, and popularity.

## Method

### Participants and Procedure

Participants were drawn from the KiVa program evaluation (see Kärnä et al., 2013). The data used in the current study included students in grade 7–9 from 78 secondary schools that were randomly assigned to either the intervention or control condition (39 schools each). Active parental consent was obtained from 87.4% of the target sample, and students were also asked to give assent before participating in the study. Four control schools dropped out before providing any data and one intervention school only participated in the first wave of data collection, which resulted in a total of 35 control schools and 38 intervention schools. For the current study, we only used data from control schools to avoid any biases between the study variables due to the intervention, and to study the effects outside of a formal, school-wide anti-bullying intervention.

Three waves of data collection occurred over the course of 1 year: May 2008 (Wave 1; grade 7–8), December 2008 (Wave 2; grade 8–9), and May 2009 (Wave 3; grades 8–9). As we were interested in examining whether defending within a specific peer group was related to adverse outcomes, the analyses for this study focused on the second and third waves of data (T1 and T2 in this study) as youth were in the same classroom at both time points. Furthermore, in the current study T1 and T2 were separated by approximately 4–5 months, as we expected any potential consequences of defending to occur within a relatively short time frame (e.g., over the span of a few months). To ensure reliability and validity of peer nomination scores, classrooms with less than 14 students and/or classrooms with a participation rate lower than 60% were excluded from the current analysis (Cillessen & Marks, 2011). The final sample consisted of 4085 students (43.9% boys; T2 *M*_age_ = 14.56, *SD* = 0.75). Most participants were born in Finland (97.0%) and 83.5% of the final sample participated in both waves of the data collection. Two-wave responders did not significantly differ from one-wave responders with one exception: two-wave responders had lower scores on depression at T2 (*t* = 2.49, *p* = 0.013) than the one-wave responders.

Students completed the online questionnaires during regular school hours. The administration of the questionnaires was supervised by teachers who received detailed instructions regarding the procedure two weeks prior to data collection. Students were assured of the confidentiality of their answers and that participation was voluntary. The order of the questions, items, and scales were randomized within the survey. This study was carried out in accordance with the Declaration of Helsinki and the recommendations of the Ethics Board of the University of Turku with written informed consent from all subjects and their parents.

### Measures

#### Defending

Defending was assessed using the Participant Role Questionnaire (PRQ; Salmivalli & Voeten, 2004), and included 3 items that described common actions that youth might engage in to comfort and defend a victimized peer (i.e., “Tries to make others stop bullying”, “Comforts the victim or encourages him/her to tell the teacher about the bullying”, “Tells the others to stop bullying”). Students could nominate an unlimited number of classmates for each item. For each participant, the received nominations were summed and divided by the number of possible nominators within each class to form a proportion score. The final defending score was created by averaging the proportion scores across the 3 items for each student, with scores ranging from 0 to 1.

#### Social Anxiety

Social anxiety was assessed using the 5-item Fear of Negative Evaluation scale (e.g., “I’m afraid the others won’t like me”; La Greca & Lopez, 1998). Students rated each statement on a 5-point scale (0 = *not at all,* 4 = *all the time*). The social anxiety scale demonstrated high internal consistency at both time points (α = 0.93 to 0.95).

#### Depressive Symptoms

Depressive symptoms were measured using a 7-item scale derived from the Beck Depression Inventory (BDI; Beck et al., 1996). Items were included based on their suitability for use with children and early adolescents. Items regarding suicide ideation and intent, sexual interest, and somatic complains were excluded. The remaining 7-item scale assessed cognitive-affective concerns (e.g., “What is your mood like?”; “How do you feel about yourself?”) and was rated on a 5-point scale, with higher values indicating higher levels of depressive symptoms. The depression scale demonstrated evidence of high reliability at both time points (*α* = 0.91 to 0.94).

#### Victimization

Both self- and peer-reports of victimization were included in the current analysis. *Self-reported victimization* was measured at T1 using the revised Olweus Bully/Victim questionnaire (Olweus, 1996). Participants completed a 10-item scale assessing how frequently they experienced different specific forms of victimization (e.g., “I was hit, kicked, or pushed”, “I was called nasty names or laughed in my face or hurt by insults”), using a 5-point scale (0 = *not at all,* 4 = *several times a week*). Participants’ responses on the 10 items were averaged to create a total self-reported victimization score (α = 0.83). *Peer-reported victimization* was assessed at T1 using 3 items from the Participant Role Questionnaire (PRQ; Salmivalli & Voeten, 2004; i.e., “s/he is called names and made fun of”, “s/he is pushed and hit”, “s/he is usually talked about with a bad tone”). Students could nominate an unlimited number of classmates for each item. For each participant, the received nominations were summed and divided by the number of possible nominators within each class to form a proportion score. The final peer-reported victimization score was created by averaging across the 3 items, with scores ranging from 0 to 1.

#### Popularity

Students’ popularity was assessed using peer nominations. Participants were asked to nominate classmates who were the “most popular”. For each student, the received nominations were summed and divided by the number of possible nominators to form a proportion score, with scores ranging from 0 to 1.

## Results

### Descriptive Statistics

Means, standard deviations, and correlations among study variables are presented in Table 1. Girls were significantly more likely to defend than boys. Girls also reported higher levels of depressive symptoms and social anxiety at both time points, whereas boys scored higher on self- and peer- reported victimization. Boys were also significantly more popular than girls.

**Table 1:** Correlations, Means, and Independent Sample T-Tests

	1	2	3	4	5	6	7	*M* (SD)	*M* (SD)_girls_	*M* (SD)_boys_	*t*
Defending T1	–							0.08 (0.10)	0.11 (0.11)	0.05 (0.06)	22.41^***^
Self-Reported Victimization T1	-0.06^***^	–						0.19 (0.43)	0.14 (0.30)	0.24 (0.54)	*-*6.52^***^
Peer-Reported Victimization T1	-0.08^***^	0.27^***^	–					0.06 (0.08)	0.05 (0.07)	0.07 (0.10)	-7.66^***^
Popularity T1	0.19^***^	-0.03	-0.14^***^	–				0.11 (0.17)	0.10 (0.17)	0.12 (0.18)	-3.27^**^
Social Anxiety T1	0.08^***^	0.13^***^	0.11^***^	-0.07^***^	–			1.47 (0.89)	1.66 (0.85)	1.25 (0.89)	14.39^***^
Depressive Symptoms T1	-0.01	0.30^***^	0.09^***^	-0.05^**^	0.37^***^	–		0.80 (0.77)	0.89 (0.78)	0.70 (0.75)	7.92^***^
Social Anxiety T2	0.08^***^	0.07^***^	0.05^**^	-0.03	0.47^***^	0.24^***^	–	1.44 (0.94)	1.63 (0.88)	1.22 (0.96)	12.54^***^
Depressive Symptoms T2	-0.02	0.22^***^	0.09^***^	-0.01	0.24^***^	0.60^***^	0.24^***^	0.80 (0.91)	0.86 (0.86)	0.73 (0.96)	4.16^***^

^**^p < 0.01; ^***^p < 0.001

Only 20.9% of the sample were not reported by any classmate as engaging in any defending. For the full sample, defending was negatively associated with self- and peer-reported victimization, and positively associated with popularity and social anxiety. There was not a significant correlation between defending and depressive symptoms at either time point. There was high stability of internalizing symptoms over time (*r* = 0.47 and *r* = 0.60 for social anxiety and depressive symptoms, respectively). Self- and peer-reported victimization were only moderately correlated at T1 (*r* = 0.27). At both time points, the correlations between victimization and depressive symptoms were stronger for self-reported victimization (*rs* > 0.22) than for peer-reported victimization (*rs* = 0.09).

### Concurrent Associations Between Defending and Internalizing Problems

 Analyses were conducted using linear regression in R. We corrected for dependencies in the data caused by students being nested in classrooms by using the “cluster” option and robust standard errors. To examine the concurrent association between defending and internalizing problems, we first examined the main effects of defending on internalizing problems (i.e., social anxiety and depressive symptoms) at T1, controlling for gender, T1 victimization, and T1 popularity (Table 2). Next, we added interactive terms between defending, popularity, and victimization to examine whether the association between defending and each internalizing problem was moderated by popularity and/or victimization. All variables included in the interactions were centered. Gender was coded as 0 = girl and 1 = boy. Separate models were conducted for self- and peer- reported victimization (see Table 2, Panels A and B).

**Table 2:** Concurrent Effects of Defending, Victimization, and Popularity on Depressive Symptoms and Social Anxiety

Panel A				
	Social Anxiety T1		Depressive Symptoms T1	
	Main Effects	Interactive Effects	Main Effects	Interactive Effects
	*b* (SE)	*b* (SE)	*b* (SE)	*b* (SE)
Gender	-0.42^***^ (0.03)	-0.42^***^ (0.03)	-0.26^***^ (0.03)	-0.27^***^ (0.03)
Defending	0.25 (0.16)	0.25 (0.18)	-0.30^*^ (0.15)	-0.28 (0.16)
Self-reported victimization	0.31^***^ (0.07)	0.37^***^ (0.07)	0.56^***^ (0.05)	0.59^***^ (0.05)
Popularity	-0.29^**^ (0.09)	-0.29^**^ (0.09)	-0.13 (0.07)	-0.13 (0.08)
Defending X Self-reported victimization	–	1.89^*^ (0.86)	–	1.12 (0.62)
Defending X Popularity	–	1.22 (0.77)	–	0.60 (0.54)
Self-reported victimization X Popularity	–	0.22 (0.29)	–	-0.23 (0.33)
Defending X Self-reported victimization X Popularity	–	6.13 (3.78)	–	5.87 (3.60)
**Panel B**				
	**Social Anxiety T1**		**Depressive Symptoms T1**	
	**Main Effects**	**Interactive Effects**	**Main Effects**	**Interactive Effects**
	*b* (SE)	*b* (SE)	*b* (SE)	*b* (SE)
Gender	-0.42^***^ (0.03)	-0.42^***^ (0.03)	-0.23^***^ (0.03)	-0.23^***^ (0.03)
Defending	0.25 (0.16)	0.23 (0.17)	-0.33^*^ (0.15)	-0.33^*^ (0.16)
Peer-reported victimization	1.46^***^ (0.19)	1.29^***^ (0.25)	1.01^***^ (0.19)	1.10^***^ (0.21)
Popularity	-0.21^*^ (0.09)	-0.26^*^ (0.10)	-0.10 (0.07)	-0.10 (0.09)
Defending X Peer-reported victimization	–	-0.09 (2.26)	–	3.40 (2.68)
Defending X Popularity	–	0.54 (0.80)	–	-0.04 (0.60)
Peer-reported victimization X Popularity	–	-2.47 (2.15)	–	-0.01 (1.54)
Defending X Peer-reported victimization X Popularity	–	9.18 (16.80)	–	-11.62 (13.87)

In Panel A, the model included self-reported victimization. In Panel B, the model included peer-reported victimization

^*^p < 0.05; ^**^p < 0.01; ^***^p < 0.001

#### Social Anxiety

The model testing the main effects of gender, defending, popularity, and victimization on social anxiety was significant for both self-reported victimization (*F*(4, 3832) = 80.89, *p* < 0.001, *R*^2^ = 0.08) and peer-reported victimization (*F*(4, 3832) = 74.94, *p* < 0.001, *R*^2^ = 0.07) (see Table 2). Girls scored higher than boys on social anxiety. The main effect of defending on social anxiety was not significant. Self-reported and peer-reported victimization were positively associated with social anxiety, whereas popularity was negatively associated with social anxiety.

**Fig. 1:**
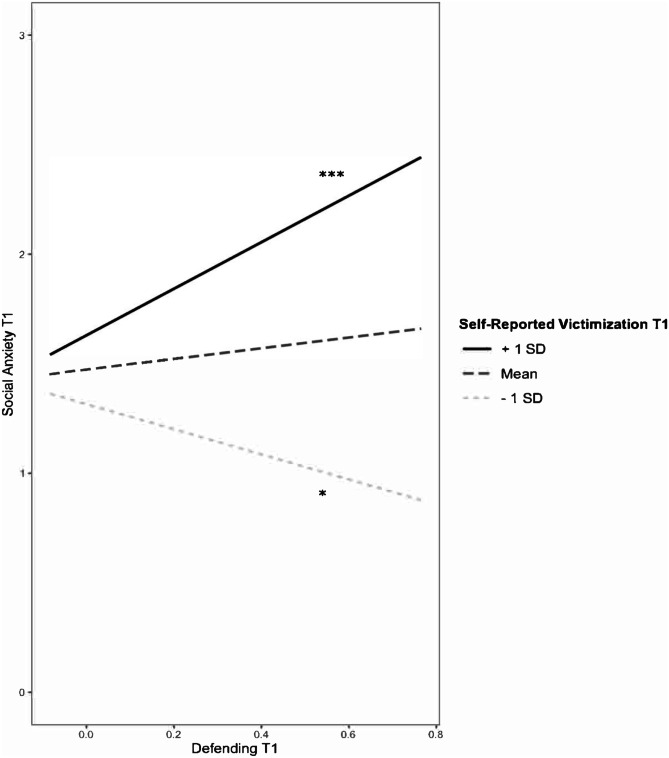
Concurrent association between defending and social anxiety moderated by self-reported victimization. *Note.* ^*^p < 0.05. ^***^p < 0.001

Next, we added the interactive terms to the models (Table 2). Again, the models were significant for both self-reported victimization (*F*(8, 3828) = 43.88, *p* < 0.001, *R*^2^ = 0.08) and peer-reported victimization (*F*(8, 3828) = 37.86, *p* < 0.001, *R*^2^ = 0.07). Adding the interactive terms resulted in a significant change in *R*^2^ for the model with self-reported victimization (*p* < 0.001) but not for the model with peer-reported victimization (*p* = 0.53). There was a significant 2-way interaction between defending and self-reported victimization in predicting social anxiety (see Fig. 1). For participants with higher levels (+ 1 SD) of self-reported victimization, defending was positively associated with social anxiety (simple slopes test, *t* = 4.35, *p* < 0.001). At lower levels (-1 SD) of self-reported victimization, defending was negatively associated with social anxiety (simple slopes test, *t* = -2.38, *p* = 0.02). The association between defending and social anxiety was not significantly moderated by peer-reported victimization. There was no evidence that the association between defending and social anxiety was moderated by popularity, and the three-way interaction between defending, victimization, and popularity was also not significant. The interaction between defending and self-reported victimization remained significant when the nonsignificant 3-way interaction was excluded from the model.

#### Depressive Symptoms

The model testing the main effects of gender, defending, popularity, and victimization on depressive symptoms was also significant for both self-reported victimization (*F*(4, 3842) = 126.65, *p* < 0.001, *R*^2^ = 0.12) and peer-reported victimization (*F*(4, 3842) = 30.01, *p* < 0.001, *R*^2^ = 0.03) (see Table 2). Girls were more likely to endorse depressive symptoms than boys. Defending was negatively associated with depressive symptoms, whereas (both self- and peer-reported) victimization was positively associated with depressive symptoms. Popularity was not significantly associated with depressive symptoms.

The models with the interaction terms added were also significant for both self-reported victimization (*F*(8, 3838) = 65.78, *p* < 0.001, *R*^2^ = 0.12) and peer-reported victimization (*F*(8, 3838) = 15.53, *p* < 0.001, *R*^2^ = 0.03). The concurrent association between defending and depressive symptoms was not significantly moderated by popularity or victimization (see Table 2). Likewise, the 3-way interaction between defending, popularity, and victimization was also not statistically significant.

### Prospective Associations Between Defending and Internalizing Problems

The longitudinal analyses were also conducted in R, using the “cluster” option and robust standard errors. To examine whether defending was associated with elevated internalizing problems over time, we first examined the main effects of defending, victimization, and popularity at T1 on internalizing problems at T2, while controlling for gender and internalizing problems at T1. Next, we added the interactive terms between defending, victimization, and popularity. Again, separate models were conducted for self- and peer- reported victimization.

#### Social Anxiety

The model testing the main effects of defending, popularity, and victimization at T1 on social anxiety at T2, controlling for gender and social anxiety at T1, was significant for both self-reported victimization (*F*(5, 3030) = 185.32, *p* < 0.001, *R*^2^ = 0.23) and peer-reported victimization (*F*(5, 3030) = 184.97, *p* < 0.001, *R*^2^ = 0.23). Social anxiety was stable over time, and girls scored higher than boys. The main effect of defending at T1 on social anxiety at T2 was not significant. When the interaction terms were added, the overall models remained statistically significant, *F*(9, 3026) > 103.14, *ps* < 0.001, *R*^2^ = 0.23; however, the changes in *R*^2^ were not statistically significant (*ps* > 0.43) and victimization and popularity were not significant moderators of the longitudinal association between defending at T1 and social anxiety at T2 (see Table 3).

**Table 3:** Prospective Effects of Defending, Victimization, and Popularity on Depressive Symptoms and Social Anxiety

Panel A				
	Social Anxiety T2		Depressive Symptoms T2	
	Main Effects	Interactive Effects	Main Effects	Interactive Effects
	*b* (SE)	*b* (SE)	*b* (SE)	*b* (SE)
Gender	-0.21^***^ (0.04)	-0.21^***^ (0.04)	-0.01 (0.03)	-0.01 (0.03)
Outcome Variable T1	0.46^***^ (0.02)	0.46^***^ (0.02)	0.70^***^ (0.03)	0.70^***^ (0.03)
Defending T1	0.13 (0.15)	0.12 (0.17)	-0.18 (0.13)	-0.11 (0.13)
Self-reported victimization T1	0.08 (0.06)	0.10 (0.07)	0.11 (0.06)	0.11 (0.06)
Popularity T1	0.02 (0.09)	0.02 (0.10)	0.22^*^ (0.10)	0.26^*^ (0.11)
Defending X Self-reported victimization T1	–	0.51 (0.70)	–	0.30 (0.63)
Defending X Popularity T1	–	0.57 (0.62)	–	-1.25 (0.68)
Self-reported victimization X Popularity T1	–	0.15 (0.50)	–	0.58 (0.46)
Defending X Self-reported victimization X Popularity T1	–	2.28 (3.74)	–	-3.41 (3.75)
**Panel B**				
	**Social Anxiety T2**		**Depressive Symptoms T2**	
	**Main Effects**	**Interactive Effects**	**Main Effects**	**Interactive Effects**
	*b* (SE)	*b* (SE)	*b* (SE)	*b* (SE)
Gender	-0.21^***^ (0.04)	-0.22^***^ (0.04)	-0.01 (0.03)	-0.01 (0.03)
Outcome Variable T1	0.46^***^ (0.02)	0.46^***^ (0.02)	0.71^***^ (0.03)	0.71^***^ (0.03)
Defending T1	0.12 (0.16)	0.11 (0.17)	-0.19 (0.13)	-0.11 (0.14)
Peer-reported victimization T1	0.35 (0.26)	0.46 (0.33)	0.33 (0.19)	0.47 (0.29)
Popularity T1	0.04 (0.10)	0.04 (0.12)	0.24^*^ (0.10)	0.27^*^ (0.13)
Defending X Peer-reported victimization	–	3.69 (2.81)	–	2.83 (1.81)
Defending X Popularity T1	–	0.15 (0.71)	–	-1.77^**^ (0.60)
Peer-reported victimization X Popularity T1	–	0.30 (2.61)	–	0.13 (2.79)
Defending X Peer-reported victimization X Popularity T1	–	-12.72 (14.96)	–	-29.54^*^ (13.54)

In Panel A, the model included self-reported victimization. In Panel B, the model included peer-reported victimization

^*^p < 0.05; ^**^p < 0.01; ^***^p < 0.001

#### Depressive Symptoms

The model testing the main effects of defending, popularity, and victimization at T1 on depressive symptoms at T2, controlling for gender and depressive symptoms at T1, was significant for both self-reported victimization (*F*(5, 3077) = 348.70, *p* < 0.001, *R*^2^ = 0.36) and peer-reported victimization (*F*(5, 3077) = 346.88, *p* < 0.001, *R*^2^ = 0.36). Depressive symptoms were stable over time. There was no significant association between defending at T1 and depressive symptoms at T2. Popularity at T1 was positively associated with depressive symptoms at T2. There were no other significant main effects.

**Fig. 2:**
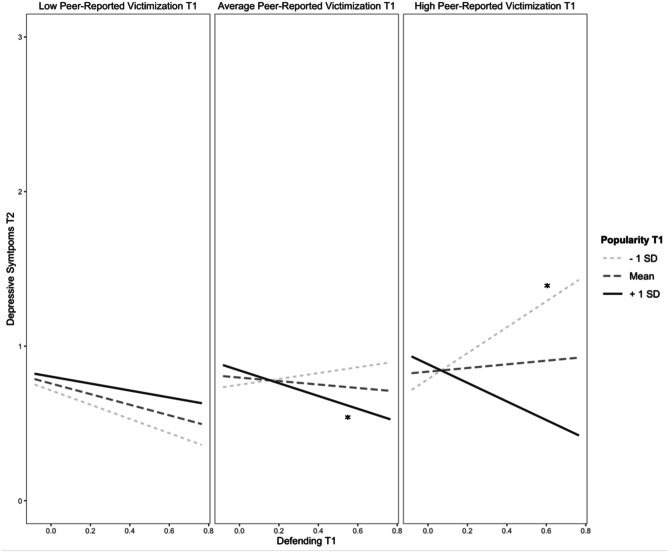
Prospective association between defending at T1 and depressive symptoms at T2 moderated by popularity and peer-reported victimization at T1. Note. ^*^p < 0.05

After adding the interaction terms, the models remained significant for both self-reported victimization (*F*(9, 3073) = 196.04, *p* < 0.001, *R*^2^ = 0.36) and peer-reported victimization (*F*(9, 3073) = 194.48, *p* < 0.001, *R*^2^ = 0.36), with a significant change in *R*^2^ for both models (*ps* < 0.02) (see Table 3). In the model with peer-reported victimization, there was a significant interaction between defending and popularity at T1 on depressive symptoms at T2. For youth with low levels of popularity at T1, defending was not significantly associated with depressive symptoms at T2 (simple slopes test, *t* = 0.92, *p* = 0.36). However, at high levels of popularity, defending at T1 was negatively associated with depressive symptoms at T2 (simple slopes test, *t* = -2.50, *p* = 0.01). In the model with self-reported victimization, this interaction did not reach significance (*p* = 0.065). However, an exploratory probe of the interaction showed the same pattern – a significant, negative slope between defending and depressive symptoms at high levels of popularity (simple slopes test, *t* = -2.04, *p* = 0.04) but not low levels of popularity (simple slopes test, *t* = 0.50, *p* = 0.62). The interaction between defending and popularity at T1 was further qualified by peer-reported victimization (see Fig. 2). At high levels of peer-reported victimization and low levels of popularity, defending at T1 was *positively* associated with depressive symptoms at T2 (simple slopes test, *t* = 2.50, *p* = 0.01). However, at average levels of peer-reported victimization and high levels of popularity, defending at T1 was *negatively* associated with depressive symptoms at T2 (simple slopes test, *t* = -2.50, *p* = 0.01).

## Discussion

Given the important role defending victimized peers may play in reducing the prevalence of bullying (e.g., Hawkins et al., 2001), the current study sought to clarify the concurrent and prospective associations between defending and internalizing symptoms, while accounting for victimization and popularity as potential moderators. Recent research has raised concerns that defending may be stressful for youth, citing positive concurrent associations between defending and internalizing symptoms (e.g., Callaghan et al., 2019; Evans et al., 2019; Lambe et al., 2017), whereas other studies have found a negative relationship (e.g., Correia et al., 2009). Thus, it remains an open question whether defending actually places youth at increased risk for experiencing negative mental health outcomes.

Our findings indicated that defending was generally *not* a risk factor for current or future internalizing problems. Overall, only limited associations between defending and internalizing symptoms were found. The main effect of defending on current symptoms of social anxiety and on future social anxiety and depressive symptoms were not significant. However, defending was negatively related to concurrent depressive symptoms after controlling for gender, victimization, and popularity. This finding is consistent with a recent meta-analysis which found that prosocial behavior is negatively associated with depressive symptoms, particularly for early adolescents (Memmott-Elison et al., 2020). Helping others has been proposed to function as a way to dispel negative arousal (Schacter & Margolin, 2018); thus, youth who defend victimized peers may experience concurrent benefits, compared to doing nothing. Conversely, youth who are higher in depressive symptoms may lack the motivational capacity to engage in prosocial behavior or to address the suffering of others. In contrast, both self- and peer-reported victimization were positively related to concurrently measured depression and social anxiety, as well increased symptoms of depression over time. The association between victimization and depressive symptom was stronger for self-reported victimization than peer-reported victimization, perhaps due to shared method variance; whereas the association between victimization and social anxiety was similar for self- and peer-reported victimization (0.13 and 0.11, respectively). Also, as expected, youth with high social status were less likely to report symptoms of social anxiety at T1; however, they reported higher levels of depressive symptoms later in the school year. This finding is consistent with previous research demonstrating that while high social status confers some social benefits, it can also be burdensome for youth (Ferguson & Ryan, 2019).

Building on these initial results, the inclusion of peer victimization and popularity as moderators of the relationship between defending and internalizing symptoms revealed that acting to defend a victimized peer may actually be beneficial for the mental well-being of some youth. Consistent with the cumulative interpersonal risk model (Epkins & Heckler, 2011), the current results suggested that having a reputation for defending was only positively associated with concurrent social anxiety for youth who were already socially vulnerable due to high levels of self-reported victimization. In fact, defending was negatively associated with social anxiety for youth with low levels of victimization. Thus, one possible explanation for the inconsistent findings in the extant literature is that most studies did not account for the overlap between defending and victimization. For youth who already feel victimized by their peers, defending was positively associated with social anxiety – perhaps as a consequence of their previous victimization, or because they are fearful of further victimization. Indeed, this perspective is consistent with the ‘retaliation hypothesis’ which suggests that youth may be reluctant to defend the victim because they are fearful of becoming the bullies’ next target if they get involved (Huitsing et al., 2014). However, it also suggests that this fear may only be true for youth who already experience relatively frequent or severe peer victimization themselves.

While these findings help clarify the concurrent association between defending and internalizing symptoms, our next goal was to examine whether defending was in fact a risk factor for the development of future mental health problems. Although the main effect of defending on later internalizing symptoms was not significant, the association between defending and subsequent depressive symptoms was moderated by popularity. Specifically, defending at T1 was negatively associated with depressive symptoms at T2 for high-status youth. Thus, defending was actually found to be protective against the development of depressive symptoms among popular youth. Higher-status youth may not only have the social resources to defend their peers without experiencing psychological distress, but it is also appears that defending may help to mitigate some of the potential risks of high popularity (e.g., poor psychosocial adjustment: Ferguson & Ryan, 2019) as defending may generate positive feedback from peers. Indeed, previous research indicates that youth who defend tend to be both popular and well-liked (Lambe et al., 2019), and their prosocial behavior is rewarded with increased social status among their peers (van der Ploeg et al., 2017).

Finally, the positive interaction between defending and popularity on subsequent depressive symptoms was further qualified by the extent to which youth were seen as a victim of bullying by their classmates. Specifically, for low-status youth with high levels of peer-reported victimization, defending positively predicted depressive symptoms over time. At the same time, defending was negatively associated with subsequent symptoms of depression among high-popular youth with average levels of peer-reported victimization. This finding provides preliminary support for both a ‘defender vulnerability’ and a ‘defender protection’ hypothesis, demonstrating that defending may have different effects on youth mental health depending on their social roles and experiences within the peer group. Moreover, consistent with past research demonstrating that victims of the same bullies are more likely to defend one another over time (i.e., the social support hypothesis; Huitsing et al., 2014), the current study confirmed that some youth do choose to defend their victimized peers even though they are also being victimized themselves. However, in the current study defending was associated with greater symptoms of depression among victimized youth, perhaps due to their pre-existing social vulnerabilities. For example, youth who are vulnerable (i.e., highly victimized with low status) but still defend others may not be able to obtain their desired outcomes from defending (i.e., defending others successfully). If the reality after defending is incongruent with their expected outcomes of defending, then this discrepancy could amplify their psychological distress. Future research is needed to further examine the mechanisms underlying the positive association between defending and internalizing symptoms for vulnerable youth.

It is important to note that while the findings were largely the same in the models using self- versus peer-reported victimization, there were a few key differences. The concurrent associations between defending and social anxiety was only significantly moderated by self-reported victimization, whereas the prospective association between defending and depressive symptoms was only significantly moderated by peer-reported victimization. Although the reasons for these differences are not yet clear, the slightly different pattern of findings for self- and peer- reported victimization underscores the importance of including both types of informants in studies when possible (Ma et al., 2019).

### Strengths, Limitations, and Future Directions

Notable strengths of the current investigation were the use of a prospective, multi-informant research design, as well as the inclusion of two potential moderators of the association between defending and internalizing symptoms (e.g., peer victimization and social status). Notwithstanding these important additions to the research literature, this study also has certain caveats and limitations, which deserve to be addressed.

First, it is possible that any internalizing problems experienced by defending result from the stress of witnessing bullying incidents and of having to decide whether to intervene or not, rather than from the actual act of defending. Future research could include a measure of witnessing bullying to further clarify the unique contributions of defending; for studies interested in the factors predicting defending, this measure would also allow a distinction between those who refrain from defending after witnessing bullying from those who do not defend simply because they have not noticed any bullying. Moreover, as defending was only positively associated with internalizing symptoms for youth high in victimization, it is possible that the decision to intervene is especially stressful for highly victimized youth who may realize they do not have the social resources to defend successfully and/or fear retaliation. Future research is needed that explores why victimized youth still choose to defend other victims, even though it may lead to additional distress. For example, victimized youth, compared to the rest of the peer group, may feel more empathy toward other victims, as they know firsthand what it feels like to be a target of peer aggression. Moreover, future research should consider potential mechanisms that could explain why defending may contribute to internalizing problems for victimized youth specifically, such as their outcome expectations and anticipated interpersonal consequences of defending.

Second, the current study does not have any information regarding whether the defending was successful. Whether or not youth obtained their desired results is an important factor for future research to consider when examining the consequences of defending for youth’s mental health. In particular, it is possible that defending may have negative implications on youth who tried to defend a victimized peer but were unable to successfully stop the bullying.

Third, defending was measured as a unidimensional construct in the current study, given that the data was collected before more recent developments in defending measurement (e.g., Bussey et al., 2020; Yun, 2020). However, a growing number of studies suggest the correlates of defending behavior differ depending on how the defending is enacted (Bussey et al., 2020; Garandeau et al., 2019; Lambe & Craig, 2020; Pronk et al., 2019; Reijntjes et al., 2016; Yun, 2020). Directly confronting bullies (e.g., assertive or aggressive defending) is likely riskier than offering support to victims (e.g., comforting defending). Youth who primarily engage in comforting defending may be less likely to experience negative consequences from defending, as they are not publicly challenging the bully and other peers may not even be aware that the defending occurred. Future research could build on the current study by examining whether the association between defending and internalizing symptoms varies depending on the type of defending youth engage in.

Lastly, there are other factors that may be relevant for whether defending leads to elevated internalizing problems. Defending may be experienced differently by youth depending on personal characteristics, such as their level of assertiveness or their feelings of self-efficacy for defending. For example, for someone who is socially withdrawn, standing up to the bully may feel more difficult. In turn, these personal traits could influence whether those who defend develop internalizing symptoms as a result. Moreover, the current study did not account for classroom-level norms that may influence whether defending is associated with risks for youth. For example, previous research has shown that youth are more likely to defend in classrooms with strong anti-bullying norms (Garandeau et al., 2019; Pouwels et al., 2019). Future research should consider how classroom-level variables, such as bullying norms, relate to the outcomes of defending.

### Conclusions

The current study did not find support for the belief that defending victims of bullying poses a risk for youth’s mental health. Rather, the findings of this study are somewhat optimistic, as defending itself was not associated with social anxiety, and was negatively related to depressive symptoms. The inclusion of peer victimization and popularity as moderators of the relationship between defending and internalizing symptoms revealed that defending may only be harmful for youth who are already vulnerable, whereas acting to defend a victimized peer can actually have a protective effect on mental well-being for youth with more social resources. Though these results require further validation, our initial results suggest that youth who are not themselves exposed to bullying by their peers could be safely encouraged to stand up for their victimized peers. We encourage further research to examine whether the psychological consequences of defending may depend on the type of defending that youth engage in (confronting vs. comforting), whether the defending was effective in helping the victim, and other personal characteristics of those who defend, as well as classroom-level social norms.
